# Complaints handling in hospitals: an empirical study of discrepancies between patients' expectations and their experiences

**DOI:** 10.1186/1472-6963-8-199

**Published:** 2008-09-30

**Authors:** Roland D Friele, Emmy M Sluijs, Johan Legemaate

**Affiliations:** 1 NIVEL, Netherlands Institute for Health Services Research, PO Box 1568, 3500 BN Utrecht, the Netherlands; 2 VU University Medical Centre Amsterdam, EMGO Institute, Amsterdam, the Netherlands; 3 Tranzo, Tilburg University, Tilburg, The Netherlands

## Abstract

**Background:**

Many patients are dissatisfied with the way in which their complaints about health care are dealt with. This study tested the assumption that this dissatisfaction consists – in part at least – of unmet expectations.

**Methods:**

Subjects were 279 patients who lodged a complaint with the complaints committees of 74 hospitals in the Netherlands. They completed two questionnaires; one on their expectations at the start of the complaints handling process, and one on their experiences after the complaints procedure (pre-post design; response 50%). Dependent variables are patients' satisfaction and their feeling that justice was done; independent variables are the association between patients' expectations and their experiences.

**Results:**

Only 31% of the patients felt they had received justice from the complaints process.

Two thirds of the patients were satisfied with the conduct of the complaints committee, but fewer were satisfied with the conduct of the hospital or the medical professional (29% and 18%). Large discrepancies between expectations and experiences were found in the case of doctors not admitting errors when errors had been made, and of hospital managements not providing information on corrective measures that were taken. Discrepancies collectively explained 51% of patients' dissatisfaction with the committee and one third of patients' dissatisfaction with the hospital and the professional. The feeling that justice was done was influenced by the decision on the complaint (well-founded or not), but also by the satisfaction with the conduct of the committee, the hospital management and the professional involved.

**Conclusion:**

It is disappointing to observe that less than one third of the patients felt that justice had been done through the complaints handling process. This study shows that the feeling that justice had been done is not only influenced by the judgement of the complaints committee, but also by the response of the professional. Furthermore, hospitals and professionals should communicate on how they are going to prevent a recurrence of the events that led to the complaint.

## Background

Many patients are dissatisfied with the way in which their complaints about health care are handled, a phenomenon that exists in a number of countries and is not well understood [1-3]. The assumption – tested in this study – is that patient dissatisfaction with complaints handling consists of unmet expectations; if patients' expectations are not met or not met in full, they may feel disappointed or even frustrated [4]. Fair complaints handling is highly significant in restoring patients' trust in health care and in renewing patients' commitment to the health care provider or organisation. Any effort to restore patient satisfaction with complaints handling is not only an ethical issue, therefore, it also provides practical advantages, while knowing the main components of dissatisfaction may lead to successful approaches to preventing dissatisfaction [5].

This article endeavours to explain patient (dis)satisfaction by comparing patients' expectations at the time they filed a complaint, with the outcome of the complaints handling process. The setting of the study was hospitals in the Netherlands and the subjects were patients who had lodged a complaint with the complaints committees of the participating hospitals.

### Patient dissatisfaction with complaints handling

Complainant dissatisfaction is a common finding in many studies [1,2,6]. Daniel evaluated the experiences of 290 patients whose complaints were finalised by the Health Care Complaint Commission of New South Wales (HCCC) and nearly two-thirds of these patients (61%) appeared to be dissatisfied with the complaints handling by the time the complaint file was closed. All but two people said they would never consult the doctor involved again, while many respondents remained angry and most wanted stern measures to be taken. Satisfaction was significantly more likely if strong action had indeed been taken against the doctor [1]. In business research, little is also known about people's behavioural and emotional responses to complaints handling [5].

### Patients' motives for complaining

An important motive for patients to lodge a complaint is to prevent the same incident from happening to others [7-9]. Complainants experienced strong feelings of having been wronged and many felt it was their duty to complain, because they experienced a sense of moral duty or justice having been violated. They reacted out of a basic feeling that something had gone wrong in how things should be arranged that had to be set right [10]. Some patients said they owed it to their deceased partner or child to lodge a complaint, while Bark et al. [7] found that the majority of patients wanted staff to be aware of what had happened and the effect it had had on the patient; "I wanted doctors to realise what they had done." [8].

### Patients' expectations of complaints handling

Some studies focused on what patients expect from the health care provider (or the accused) in response to their complaint. According to Gallagher, patients want full disclosure of the incident [11], which is likely to increase patient satisfaction [12]. Bark et al. [7] and Vincent et al. [8] found that many patients wanted an explanation from their doctor and/or a detailed account of what had happened. Half of the patients wanted an apology (as well), while less than 10% of the complainants attempted to obtain financial compensation [6,7]. Bismark et al. [9] found that most patients do not seek monetary compensation when given a choice, but appreciate other forms of accountability, like explanations or lessons learned. They state that "the offering of apologies, explanations and assurances of system change, where appropriate, may address many patients' true concerns...". Only in the case of patients who had sued their doctor, did two thirds want to receive financial compensation [8].

Complaints are handled by patient ombudsmen or (hospital) complaints committees in some countries. Committees of this kind, which are required by law in the Netherlands, act as intermediaries between the hospital and the complainant and are responsible for a careful and independent handling of patients' complaints [13,14]. What patients expect of the complaints committee differs from what they expect of the hospital or the doctor who gave rise to the complaint [4].

### Hypothesis and research questions

The study is based on the underlying hypothesis that patient satisfaction with complaints handling is based on the extent to which their expectations regarding the conduct of the committee, the hospital and the professional are met. This hypothesis will be tested by answering the following research questions in succession.

1. What is the association between patients' initial expectations of the complaints handling process and their final experiences of the complaints committee, the medical professional, and the hospital management?

2. What factors in the conduct of the committee, the professional, and the hospital management predict patients' satisfaction with the complaints handling process?

3. Does satisfaction with complaints handling contribute to the feeling that justice was done through the complaints handling procedure?

The hypothesis on fair complaints handling stems from the core concepts in both Fairness and Justice Theories [4,15]. According to these theories, patient satisfaction depends on the perceived fairness of a) the complaint procedures, b) the interpersonal communication, and c) the outcome.

The fairness of the complaints procedures is related to procedural justice, which is mainly expected of the complaints committee [10]. The fairness of the communication is related to interpersonal behaviour, treating people with dignity and respect for example, i.e. interactional justice, which is expected of the members of the committee and the medical professional as well [10]. The fairness of the outcome relates to the final decision, or what the patient "gets out of" the complaints handling process, i.e. distributive justice. Three types of outcome can be distinguished, viz. the committee's decision on the complaint, the doctor's explanation or apology, and the hospital's corrective measures or changes [10].

Fair outcomes alone do not determine patient satisfaction. It is often *how *(in terms of process and interpersonal style) the outcome is communicated, rather than *what *is communicated that seems to matter [15], which means that interpersonal communication plays a dominant role in a person's decision to remain loyal or to discontinue the relationship [5]. Satisfaction will also depend on the remedial options available, which are naturally restricted in health care, where it usually is not possible to undo what has happened to the patient. Patients will judge fairness against the efforts of the hospital to make amends for the incident [15]. If people feel that feasible remedial options exist (changes at the hospital, for example), but the provider does not use any of these options, the provider will be perceived as not caring and not caring is likely to evoke negative emotions and to result in anger and dissatisfaction [15].

## Methods

### Design of the study

In order to assess expectations of the complaints procedure, patients received a first questionnaire (pre-test) via the complaints committee immediately after they had lodged a complaint. They then returned the questionnaires directly and anonymously to our research institute (NIVEL). Only complainants who had completed an informed consent form (revealing their name and address to the researchers) subsequently received a second questionnaire (post-test) directly from our research institute about 5 months after the first one, in order to assess their evaluation of the complaints procedure.

### Privacy protection

Before taking up this study the need for formal ethical approval was considered. After consulting an external medico-legal adviser it was concluded that under Dutch law formal ethical approval for this study was not required, since it does not concern a medical intervention. Also the impact of the questionnaires on daily life was considered minor [16,17]. Apart form this decision a privacy protocol was drafted. It consisted of the following elements.

- Neither the complaints committees nor the hospitals saw the completed questionnaires and neither party knew which patients participated in the study.

- It was explained in a letter that the complainants were entirely free to decide whether or not to complete the questionnaire; no reminder would follow.

- It was explained in a letter that patients' responses to the questionnaire would and could have no bearing on the conduct or outcome of the complaints procedures.

- Complainants' responses were treated confidentially. A written privacy protocol was used to process the data.

### Questionnaire

Two questionnaires were developed, based on the patients' perspective as derived from interviews and focus group meetings with complainants [10]. The following items were included, in addition to demographic questions.

Pre-test:

- The consequences i.e. impact of the complaint.

- Patients' initial expectations regarding the complaints committee, the hospital and the medical professional who gave cause for the complaint.

Post-test:

- Patients' experiences of the conduct of the complaints committee and of the reactions of the hospital and the medical professional.

- Patients' satisfaction with a) the committee, b) the hospital and c) the medical professional, patients' overall feelings that justice was done (dependent variables) and the decision of the committee on the complaint.

Both questionnaires contained similar closed questions on patients' expectations and their experiences respectively (for items see tables 2 and 4). The items relating to patients' expectations had four response categories, i.e. For me this issue is: 1 = not important; 2 = important; 3 = very important; 4 = extremely or most important. Patients could tick whether or not the complaint had impact in up to three areas, viz. physical suffering, mental suffering, financial consequences (maximum score of impact = 3).

Post-test, patients' experiences regarding the same items could be ticked on a four-point scale: really achieved, achieved, not achieved, really not achieved. Patient satisfaction with the conduct of the committee, the hospital management and the relevant professional was rated on a four-point scale (definitely satisfied, moderately satisfied, moderately dissatisfied, definitely dissatisfied) and the patient's overall feeling that justice had been done was rated as yes, to some extent, or no. Respondents also reported the decision of the complaints committee.

### Respondents

The complaints committees of all 97 hospitals were invited to take part in the study and 76 of them participated (response 78%). The pre-test questionnaire was sent to 563 complainants and 424 (75%) were returned. Of these 424 complainants, 67% were female and 40% had a higher professional or university education, 68% of complaints concerned the medical treatment. Of the 424 respondents, 376 returned the informed consent form as well. The post-test questionnaire was only sent to these 376 complainants, 279 (50% of 563) of whom responded. This article is based on the 279 paired pre-test/post-test questionnaires.

### Analyses

Descriptive statistics were used to compare patients' initial expectations with their final experiences. An association score was subsequently calculated, by taking the importance of an item for that particular patient (ranging from 1 to 4) multiplied by whether or not the item was achieved (recoded to -1 ((really) not achieved) and +1 ((really) achieved). For example: An extremely important item (value 4) that was achieved (value 1), was coded +4. An extremely important item (value 4) that was not achieved (value -1), was coded -4. The relationship between patients' satisfaction and the association score was analysed in three separate multiple linear forward stepwise regression analyses. The dependent variables are patients' satisfaction with a) the complaints committee, b) the hospital, and c) the medical professional. Independent variables are demographic characteristics (age, gender, education), the severity of the complaint and the association scores between expectations and experiences. A final linear regression analysis related patients' satisfaction to their overall feelings that justice had been done and the decision of the complaints committee was also included in this analysis.

## Results

### The complainants

Of the 279 responding complainants 65% were female and the mean age was 52 years (range 19 – 83). The respondents represented a relatively high educational level: 43% had higher professional or university education. The event that gave cause to the complaint usually had several medical and interpersonal aspects. Two thirds of the complaints (66%) concerned medical treatment, often in combination with shortcomings in interpersonal or informational conduct (57% and 41% respectively). These characteristics are similar to those of the population at t = 0 (n = 424). Nearly all complainants considered the incident to be serious and many reported physical and/or mental suffering (82% and 85% respectively), or financial consequences (64%). A minority of the complainants (8%) had made a claim for financial compensation (Table 1).

**Table 1:** Population characteristics (N = 279)

**Demographic characteristics**	
Females	65%
With higher professional or university education	43%
Age, mean	52 yrs
Age, range	19 – 83 yrs

**Complaint characteristics**	

Concerned medical treatment	66%
Concerned nursing care	22%
Concerned lack of information	41%
Concerned interpersonal conduct	57%
Concerned organisation of care	38%

**Reported impact of event giving rise to complaint**	

Gave rise to physical discomfort, pain or handicap	82%
Gave rise to mental suffering	85%
Had financial consequences	64%

**Made a claim for financial compensation**	

Made a claim for financial compensation	8%

**Table 2:** Comparison between patients' initial expectations and patients' final experiences of the complaints committee, expressed as percentages of patients (N = 279)

***Procedural *** **conduct**	**Expectations ** very/most important %	**Experiences** % of patients who considered the item very/most important and reported that their expectations were not met
- recommendations to the hospital to make changes	94	53
- decision on the validity of the complaint	83	16
- rationale for the decision	82	42
- investigation into the incident	80	35
- clear information about the complaints procedures	61	31
- opportunity to give a personal account of what happened	57	30
- swift response	45	43

***Interpersonal conduct***		

- impartial attitude and position	92	36
- respectful treatment	84	21
- patient's account of what had happened was listened to	75	23
- understanding shown for the patient's experiences	74	37
- sympathy shown for what the patient had been through	47	49

### Patients' experiences with the complaints committee

Hospital complaints committees in the Netherlands are required by law to work in compliance with transparent written procedures and to give a well-reasoned decision on whether a complaint is well-founded or unfounded. Table 1 shows patients' experiences with the conduct of the complaints committee compared with the issues that patients considered to be very or most important at the start of the complaints process. The items are ranged under two themes, viz. the committee's procedures and the interpersonal communication.

The issue considered to be most important by 94% of the patients at the start of the complaints procedure was a recommendation from the committee to the hospital to change things in response to the complaint. More than half (53%) of those patients who found this issue important, reported that the committees had not made such a recommendation. Making recommendations showed the greatest discrepancy between patients' expectations and the outcomes. Furthermore, 42% of the patients who found it important to receive a rationale for the committees' decision said they were not provided with such an explanation. One issue in the interpersonal conduct of the committee gave rise to disappointment for patients. The committee's impartial attitude was considered most important by nearly all patients (92%), but one third of the patients that considered this issue important did not feel that the committee demonstrated this impartial attitude. Respectful treatment by the members of the committee and listening to the patient's account of what had happened are more in line with patients' expectations (Table 2).

The complaints committee decided 31% of the complaints were well-founded, 36% well-founded in part, 21% were judged as unfounded and 1% of the complaints were dismissed. The "other" patients (11%) did not tick this item in the questionnaire and many of them described their experiences in their own words, which could not be coded in one of the earlier categories (Table 3).

**Table 3:** Decision of the complaints committee on the complaint

Decision on the complaint	%
- well-founded	31
- well-founded in part/unfounded in part	36
- unfounded	21
- dismissed	1
- other/missing	11

**Table 4:** Comparison between patients' initial expectations and patients' final experiences of the responses of the hospital management and the medical professional expressed as percentages of patients (N = 279)

***Hospital management***	**Expectations** very and most important %	**Experiences** % of patients who considered the item very/most important reporting that their expectations were not met
- ensure the complaint is discussed with the employees or department involved	86	61
- inform me **that **corrective measures have been taken	84	77
- inform me **which **corrective measures have been taken	73	88

***Medical professional(s)***		

- admit an error if an error was made	89	79
- explain how the incident could have happened	75	79
- offer an apology	45	81
- show sympathy for what I went through	44	80
- make an effort to restore our relationship	19	88

### Patients' experiences with the hospital and professionals

Hospitals in the Netherlands are legally obliged to inform the complainant whether or not (corrective) measures will be taken as a result of the complaint, and which measures will be taken. Table 4 shows patients' experiences with the responses of the hospital and the medical professional.

There are major discrepancies between patients' initial expectations and their experiences of the hospital management and the medical professional. Many patients expected the hospital management to take corrective measures in response to the complaint and/or to discuss the incident with the employees or department involved, but many patients stated this had not happened. A majority (77%) of patients who considered it important, said they were not informed by the hospital management about changes or measures within the hospital, whereas 84% of the patients considered such changes most important.

Where the medical professional who gave cause for the complaint was concerned, many patients (89%) expected to hear an admission of the error if an error had been made (as was literally stated in the questionnaire). This subject produces the greatest discrepancy between patients' expectations and their experiences. The incident was disclosed to 21% of the patients who considered this important, and about the same proportion of patients who found this important received an explanation and/or apology for the incident.

### Patients' satisfaction and the feeling that justice was done

Patients were asked (in the questionnaire) if they were satisfied with the way in which their complaint had been handled by the complaints committee, the hospital and the medical professional. The majority of patients (63%) were satisfied with the way in which their complaint had been handled by the complaints committee. Fewer were satisfied with the responses of the hospital and the doctor; 71% (60% + 11%) of the patients were dissatisfied with the response of the hospital management and 82% (71% + 11%) with the reactions of the professional who gave cause for the complaint (Table 5).

**Table 5:** Patients' satisfaction with the conduct of the complaints committee, the hospital management and the medical professional (%) (N = 279)

	complaints committee	hospital management	medical professional
Definitely satisfied	39	14	8
Moderately satisfied	24	15	10
Moderately dissatisfied	11	11	11
Definitely dissatisfied	26	60	71

**Figure 1:**
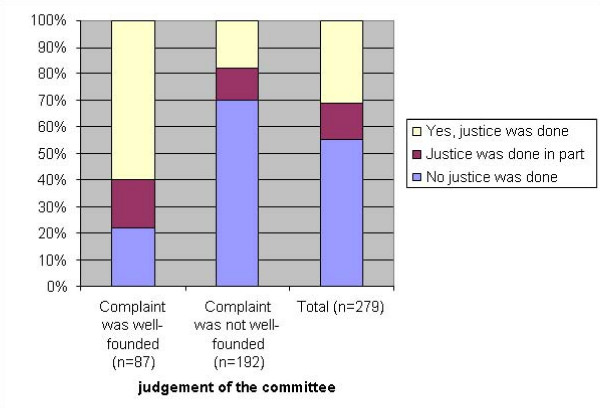
Patients' feeling that justice had been done by the decision on the complaint (n = 279).

Finally, only 31% felt that justice had been done, a feeling that is related to the judgement of the committee. Of the patients with a well-founded complaint, 60% felt that justice had been done, whereas only 18% of the patients with a complaint that was not considered well-founded or partially founded felt that justice had been done (figure 1).

### Unmet expectations and patient satisfaction

This paragraph explores the relationship between patient satisfaction with complaints handling and the degree to which expectations were met. The degree to which expectations were met was based on the association between a patient's initial expectations and final experiences and was calculated per item (see methods). Multiple linear regression analyses were used to relate association scores to patients' satisfaction with the complaints committee, the hospital and the medical professional respectively. The correlation of the association scores ranged from -0.02 to 0.66. Two items with a correlation of over 0.6 were dropped from the analyses to avoid problems of co-linearity. The impact of the complaint and the patient's demographic characteristics (gender, age and education) were included in the analyses. The results of the analyses are reported in Table 6, which shows the variables in the final model of a stepwise regression analysis and also shows the variables that were not included because they did not contribute significantly to the model (p <.05).

**Table 6:** The relationship between the association between patients' expectations and experiences on the one hand and patient satisfaction with complaints handling on the other hand (N = 279)

*Independent variables*	
***Dependent variable: Satisfaction with complaints committee***	**adjusted R^2 ^= 0.51**

Independent variables **in **the equation	*Beta (standardised) (p-value)*
- Committee showed an impartial attitude	0.45 (0.000)
- Committee showed sympathy for what the patient had been through	0.13 (0.013)
- Committee gave clear information about the procedures	0.14 (0.006)
- Committee made recommendations to hospital to make changes	0.16 (0.002)
- Committee responded swiftly	0.11 (0.032)
- Gender of patient (male (1), female (2))	0.14 (0.003)
- Impact of the incident underlying the complaint (0 = no harm to 3 = harm in three areas)	- 0.10 (0.037)

Independent variables **not **in the equation	*B if put in model (p-value)*

- Respectful treatment by committee	0.07 (0.195)
- Committee listened to patient's account of what had happened	0.10 (0.062)
- Committee investigated the incident	0.10 (0.068)
- Committee made a decision on the validity of the complaint	-0.02 (0.678)
- Committee explained the rationale for the decision	0.04 (0.480)
- Committee gave the opportunity to provide a personal account of what had happened	-0.01 (0.813)
- Level of education (1–5)	0.01 (0.907)
- Age (years)	-0.02 (0.686)

***Dependent variable: Satisfaction with hospital management***	***adjusted *R^2 ^= 0.31**

Independent variables **in **the equation	*Beta (standardised) (p-value)*

- Hospital discussed the complaint with employees	0.42 (0.000)
- Hospital reported what corrective measures had been taken	0.20 (0.001)
- Impact of the incident underlying the complaint (0 = no harm to 3 = harm in three areas)	- 0.16 (0.004)
- Gender of patient (male (1), female (2))	0.11 (0.045)

Independent variables **not **in the equation	*B if put in model (p-value)*

- Level of education (1–5)	0.10 (0.087)
- Age (years)	0.01 (0.904)

***Dependent variable: Satisfaction with medical professionals***	***adjusted *R^2 ^= 0.33**

Independent variables **in **the equation	*Beta (standardised) (p-value)*
- Professional showed sympathy for what patient went through	0.21 (0.004)
- Professional offered an apology	0.22 (0.002)
- Professional explained how things had happened	0.15 (0.022)
- Professional made efforts to restore the relationship	0.17 (0.010)
- Impact of the incident underlying the complaint (0 = no harm to 3 = harm in three areas)	-0.14 (0.013)

Independent variables **not **in the equation	*B if put in model (p-value)*

- Professional admitted an error if an error was made	0.10 (0.153)
- Gender of patient (male (1), female (2))	0.61 (0.280)
- Level of education (1–5)	-0.00 (0.949)
- Age (years)	-0.05 (0.420)

Patients' satisfaction with the complaints committee is strongly related to the committee's procedures and interpersonal behaviour, explaining 51% of the total variance. The most important of these factors, having the highest standardised beta value, is the association score of the impartial attitude of the committee. Making recommendations to the hospital, showing sympathy, transparent complaint procedures and a swift response also contribute to satisfaction with the committee. Female complainants were relatively more satisfied than male ones. The standardised beta scores can be used to compare the relative importance of the various independent variables. To give insight in the absolute impact of the independent variables we provide the following example. The (not standardised) beta value for showing an impartial attitude in this analysis is 0.16 (not in table), implying that a 1 unit change in the association score (which ranges from -4 to + 4) corresponds with a 0.16 increase in satisfaction (which ranges from 1 to 4).

Patients' satisfaction with the hospital is partly explained by association scores for two actions taken by the hospital management, i.e. informing the patient that the complaint had been discussed with the employees or department involved (most important) and that corrective measures had been taken. Although most patients did not hear about such measures, those patients who were informed about them were more satisfied than patients who remained uninformed. Female complainants were relatively more satisfied than male ones. A total of 31% of the variance in satisfaction with hospital management could be explained by these variables.

Association scores of four actions by the professional were independently related to patients' contentment. Patients were more satisfied when the professional offered an apology and showed sympathy for what had happened, when the professional explained how it had been possible for the incident to happen, and when some efforts were made to restore the doctor-patient relationship. Interpersonal behaviour of this kind on the part of the doctor in response to the complaint explains one third of the variance in patients' satisfaction.

The impact of the complaint mattered in all analyses, with high impact complaints meeting with lower patient satisfaction with the conduct of the committee, hospital management and the medical professional.

A final regression analysis was made to assess the relationship between patients' satisfaction and their feelings that justice had been done (Table 7). We saw earlier that the feeling of justice was influenced by the decision of the committee and we therefore tested the relationship between patient satisfaction and the decision of the committee in one combined analysis of the feeling that justice had been done. We found that 42% of the feeling that justice had been done could be explained by these four variables. All four contribute significantly to the explanatory power and the decision of the committee alone explained 21% of all variance (adjusted R^2^).

**Table 7:** The relationship between patient satisfaction with complaints handling by the committee, the hospital management and the professional and the decision on the complaint on the one hand, and the feeling that justice had been done on the other hand (N = 279)

**Dependent variable: feeling that justice had been done**	**adjusted R^2 ^= 0.42**
*Independent variables*	*Beta (standardised) (p-value)*

Satisfaction with complaints handling by Committee	0.32 (0.000)
Satisfaction with complaints handling by Hospital management	0.15 (0.009)
Satisfaction with complaints handling by Professional	0.21 (0.000)
The decision on the complaint (1 = complaint was well-founded, 0 = other decisions)	0.22 (0.000)

## Discussion

### What this study sought to explain

This study sought to find explanations for patients' dissatisfaction with complaints handling in hospitals, the assumption being that dissatisfaction consists – partly at least – of patients' unmet expectations. This assumption was tested by comparing a patient's expectations at the start of the complaints handling process with what they had achieved afterwards at the completion of this process and their satisfaction with the process of complaints handling. The study is unique as such, because patients' expectations were assessed before the complaints handling process had started and were linked to the outcome of the procedure.

### Three significant parties in complaints handling

The results show that many patients were quite satisfied with the way in which the committee had dealt with their complaint. Most patients, however, were dissatisfied with the reactions of the hospital and the professional in response to the complaint, specifically with the lack of response from these parties. This difference may partly be caused by the different roles of these parties. The committee was not involved in the occurrence of the event that triggered the complaint. The professionals, and to a lesser extent the hospital management, were and this may have caused the differences in satisfaction with these parties. Other more specific factors related to their conduct in the process of complaints handling also explain differences in satisfaction.

Only one third of the complainants felt that justice had been done through the complaints handling procedure, a feeling that is influenced by the decision about the complaint (well-founded or not), but also by satisfaction about the conduct of all three parties involved, viz. the committee, the hospital management and the professional. Each has a unique impact on the feeling that justice was done.

### The complaints committee

The assumption that unmet expectations are an important aspect of dissatisfaction was confirmed to some extent with regard to patients' experiences with the complaints committee. Patients' satisfaction could be explained to a great extent (51%) by the discrepancy between patients' initial expectations and their experiences with the conduct of the complaints committee. The major factor was the perceived impartiality of the committee, followed by the transparency of the procedures, the swiftness of a response, and the willingness of members of the committee to listen to the patient's story. These findings are in line with fairness and justice theories, which predict that patients' overall judgement of fairness will depend on fair procedures and fair interpersonal communication. The relative effects that these three aspects of fairness have on satisfaction are far from clear as yet [5,18], although our results suggest that satisfaction with the committees' conduct might be primarily determined by their procedural fairness, i.e. by the degree to which they demonstrate impartiality.

### The hospital management

A minority of patients was satisfied with the reactions of the hospital management. Most patients expected the hospital to take corrective measures in response to the complaint and that the management would ensure the complaint was discussed with the employees or the department involved, but most patients reported that no such action was undertaken in the hospital. Nearly one third of patient dissatisfaction could be explained by the discrepancy between patients' expectations and their achievements. Two comments would seem appropriate in this context. A hospital faced with complaints may be expected to discuss them with the employees, which may even be the natural and customary procedure in the light of current quality management and/or risk management in hospitals [19]. The problem here seems to be that most patients remained uninformed about such measures and this lack of information might contribute to the patients' negative emotions, as is predicted by fairness theory. Patients will perceive the hospital as not caring, since feasible remedial options exist, but the hospital fails to use them. When a patient perceives a hospital as not caring, however, this will give rise to frustration or even anger [15]. The obvious recommendation is to let patients know that the complaint was discussed with the persons involved.

### The professional

Many patients were disappointed in the reactions of the professional in response to their complaint and they did not achieve what they had expected. Their expectations are related to fair interpersonal communication and the greatest discrepancy between patients' expectations and their achievements concern disclosure by the professional. The majority of complainants considered it very important to hear the professional admit an error if an error had been made, but this seldom happened. Two barriers may have prevented the professional from making a full disclosure, however. Firstly, the professional and the patient may have different opinions regarding the incident. What a patient believes to have been an error may not have been perceived as such by the professional, or may have been caused by a chain of events. The second barrier is more serious, however, and involves liability. Fear of liability appears to prevent professionals from disclosing errors [11,20] and hospital insurance companies in the Netherlands explicitly forbid medical professionals to acknowledge culpability. Although professionals are allowed to give an explanation of what happened, they must refrain from any accountability [21]. This problem is the subject of an ongoing legal and ethical debate between insurance companies and professional organizations of medical doctors. Complainants not only want professionals to offer an apology, they want them to do more as well, viz. to offer sympathy and an explanation of how things had happened. This explanation, however, may prove to be the most difficult objective to achieve. Dealing with patients who file a complaint should be made part of medical training [21] and openness in the doctor-patient relationship might be facilitated by a "no fault" compensation scheme [22]. A similar issue arises in the field of patient safety. If patient safety is to be improved, there must be openness on situations where things went wrong, where patient safety was at risk. Openness is needed in order to learn from such incidents and to make improvements, but this openness is not easily achieved. The issue of patient safety is a high priority issue among Dutch policy-makers and among doctors, which has had the effect of increasing openness on the part of the medical professionals with regard to situations where patient safety was at risk [23]. This process may work as leverage to increase the openness of professionals towards complaints from patients as well.

### Strengths and weaknesses of the study

One strength of the study is its pre-post test design. The response at follow-up is 52%, which is not really high and there is no way of knowing whether the non-respondents' experiences with the complaints handling procedure were identical to the experiences of those that did respond. We noticed great similarity, however, in the socio-demographic characteristics of the population at t = 1 and at t = 2. A second strength of the study is the way in which patients' expectations were assessed. The questionnaire asked what patients considered to be important to them, i.e. what they believed should happen, which means that patients' expectations reflect their needs and demands more than what they think will happen. The study focused intentionally on complaints handling from the patient's perspective. This may be considered to be a strength of the study, but the absence of the perspectives of the committee, the hospital and the doctor may be considered to be a weakness.

## Conclusion

Our results suggest that complaints handling might become less frustrating for complainants if the complaints committee were to sit down with a complainant at the start of the process and find out what the complainant's own particular needs are, so that these needs can subsequently be addressed during the complaints handling process. Our results also show that some expectations are shared by many complainants and that complaints handling could be improved for complainants in general by meeting these expectations, which are impartiality on the part of the committee, offering apologies and informing complainants about lessons learned.

## Competing interests

The authors declare that they have no competing interests.

## Authors' contributions

RF and ES conceived and carried out the study. RF drafted the manuscript, JL provided the medico legal discussion. All authors read and approved the final manuscript.

## Pre-publication history

The pre-publication history for this paper can be accessed here:


